# Effectiveness of a family-, school- and community-based intervention on physical activity and its correlates in Belgian families with an increased risk for type 2 diabetes mellitus: the Feel4Diabetes-study

**DOI:** 10.1186/s12889-020-09336-7

**Published:** 2020-08-12

**Authors:** Nele Huys, Vicky Van Stappen, Samyah Shadid, Marieke De Craemer, Odysseas Androutsos, Katja Wikström, Konstantinos Makrilakis, Luis A. Moreno, Violeta Iotova, Tsvetalina Tankova, Anna Nánási, Yannis Manios, Greet Cardon, on behalf of the Feel4Diabetes-study group, Yannis Manios, Meropi Kontogianni, Odysseas Androutsos, George Moschonis, Konstantina Tsoutsoulopoulou, Christina Mavrogianni, Christina Katsarou, Eva Karaglani, Eirini Efstathopoulou, Ioanna Kechribari, Konstantina Maragkopoulou, Effie Argyri, Athanasios Douligeris, Mary Nikolaou, Eleni-Anna Vampouli, Katerina Kouroupaki, Roula Koutsi, Elina Tzormpatzaki, Eirini Manou, Panagiota Mpinou, Alexandra Karachaliou, Christina Filippou, Amalia Filippou, Jaana Lindström, Tiina Laatikainen, Katja Wikström, Karoliina Nelimarkka, Jemina Kivelä, Päivi Valve, Greet Cardon, Julie Latomme, Vicky Van Stappen, Nele Huys, Lieven Annemans, Lore Pil, Peter Schwarz, Ivonne Panchyrz, Maxi Holland, Patrick Timpel, Konstantinos Makrilakis, Stavros Liatis, George Dafoulas, Christina-Paulina Lambrinou, Angeliki Giannopoulou, Lydia Tsirigoti, Evi Fappa, Costas Anastasiou, Konstantina Zachari, Lala Rabemananjara, Dimitrios Kakoulis, Mayur Mandalia, Maria Stella de Sabata, Niti Pall, Luis Moreno, Fernando Civeira, Gloria Bueno, Pilar De Miguel-Etayo, Esther Ma Gonzalez-Gil, Maria I. Mesana, Germán Vicente-Rodriguez, Gerardo Rodriguez, Lucia Baila-Rueda, Ana Cenarro, Estíbaliz Jarauta, Rocío Mateo-Gallego, Violeta Iotova, Tsvetalina Tankova, Natalia Usheva, Kaloyan Tsochev, Nevena Chakarova, Sonya Galcheva, Rumyana Dimova, Yana Bocheva, Zhaneta Radkova, Vanya Marinova, Imre Rurik, Timea Ungvari, Zoltán Jancsó, Anna Nánási, László Kolozsvári, Remberto Martinez, Marcos Tong, Kaisla Joutsenniemi, Katrina Wendel-Mitoraj

**Affiliations:** 1 Department of Movement and Sport Sciences, Ghent University, Watersportlaan 2, 9000 Ghent, Belgium; 2 Department of Endocrinology and Metabolic Diseases, Ghent University Hospital, Corneel Heymanslaan, 10 Ghent, Belgium; 3 Department of Rehabilitation Sciences, Ghent University, Corneel Heysmanslaan, 10 Ghent, Belgium; 4 Research Foundation Flanders, Egmontstraat 5, Brussels, Belgium; 5 Department of Nutrition and Dietetics, School of Health Sciences & Education, Harokopio University, El. Venizelou 70, Kallithea, Athens, Greece; 6 Department of Public Health Solutions, National Institute for Health and Welfare, Mannerheimintie, 166 Helsinki, Finland; 7 First Department of Propaedeutic Internal Medicine, National and Kapodistrian University of Athens, 75 Mikras Asias str, Athens, Greece; 8 Growth, Exercise, Nutrition and Development (GENUD) Research Group, Instituto Agroalimentario de Aragón (IA2), Instituto de Investigación Sanitaria Aragón (IIS Aragón), University of Zaragoza, Calle Pedro Cerbuna, 12 Zaragoza, Spain; 9 Department of Paediatrics, Medical University of Varna, 55 Marin Drinov str, Varna, Bulgaria; 10 Clinical Center of Endocrinology, Medical University of Sofia, Boulevard “Akademik Ivan Evstratiev Geshov, 15 Sofia, Bulgaria; 11 Department of Family and Occupational Medicine, University of Debrecen, Egyeterm tér 1, Debrecen, Hungary

**Keywords:** Type 2 diabetes mellitus, High-risk families, Primary schoolchildren, Parents, Healthy lifestyle promotion, Intervention effectiveness

## Abstract

**Background:**

The study aimed to investigate the effectiveness of the European Feel4Diabetes intervention, promoting a healthy lifestyle, on physical activity and its correlates among families at risk for type 2 diabetes mellitus (based on the Finnish Diabetes Risk Score) in Belgium.

**Methods:**

The Feel4Diabetes intervention involved three components: family, school and community component, with the family component consisting of 6 counseling sessions for families at risk. Main outcomes were objectively measured physical activity levels and its subjectively measured correlates. The final sample consisted of 454 parents (mean age 39.4 years; 72.0% women) and 444 children (mean age 8.0 years; 50.1% girls). Multilevel repeated measures analyses were performed to assess intervention effectiveness after 1 year.

**Results:**

In parents, there was no significant intervention effect. In children, there were only significant negative effects for moderate to vigorous physical activity (*p* = 0.05; η_p_^2^ = 0.008) and steps (*p* = 0.03; η_p_^2^ = 0.006%) on weekdays, with physical activity decreasing (more) in the intervention group.

**Conclusions:**

The F4D-intervention lacks effectiveness on high-risk families’ physical activity and its correlates in Belgium. This could partially be explained by low attendance rates and a large drop-out. To reach vulnerable populations, future interventions should invest in more appropriate recruitment (e.g. more face-to-face contact) and more bottom-up development of the intervention (i.e. co-creation of the intervention with the target group).

**Trial registration:**

The Feel4Diabetes-study was prospectively registered at clinicaltrials.gov as NCT02393872 on 20 March 2015.

## Background

The worldwide prevalence of diabetes mellitus is rapidly increasing. In Europe, it is estimated to rise from 9.1% in 2017 to 10.8% of adults (20–79 years old) in 2045 [1]. Specifically in Belgium, 6.8% of adults was living with diabetes mellitus in 2017 [1], 87–91% of which concerning type 2 diabetes mellitus (T2DM) [2]. A major concern is that T2DM-prevalence is also increasing among children and adolescents [3]. Although prevalence numbers in Europe are scarce, evidence from the United Kingdom shows an incidence of 0.53 per 100,000 children (< 15 years old) per year [4]. Furthermore, data from Germany and parts of Austria showed that T2DM incidence in 10- to 20-year olds increased from 0.8% in 1996 to 3.3% in 2003 [5]. As T2DM has significant financial and health impacts (e.g. cardiovascular disease, blindness, kidney failure) [3], preventive strategies are needed to tackle the rising prevalence.

Since about 88.0% of children (< 20 years old) diagnosed with T2DM have a family history of the disease, targeting families, i.e., both parents and their children [6] may be a good and cost-effective strategy for T2DM prevention efforts. Furthermore, it seems of utmost importance to prioritize families at high risk for T2DM development to increase cost-effectiveness [7]. Evidence shows that having a low educational level and being unemployed are associated with a 45 and 31% increase in the risk of T2DM, respectively [8], which could partially be explained by a higher prevalence of modifiable risk factors of T2DM like an unhealthy diet or less physical activity (PA) [9].

As overweight and obesity play an important role in the development of T2DM in both adults and children, they are key factors in the prevention of T2DM [10, 11]. Next to a healthy diet, the promotion of PA is essential in the prevention of overweight, obesity and T2DM, which was also stated in the IMAGE Toolkit (a European Guideline and Training Standards for Diabetes Prevention) [12]. T2DM prevention interventions should therefore aim to increase PA levels, especially in families with an increased risk to develop T2DM.

Many factors on several levels (i.e. personal, social and environmental level) play an important role in the determination of PA behavior [13], and it is therefore necessary that PA-promoting interventions use a multi-level approach, integrating the personal and environmental level [14]. Several (systematic) reviews have shown that a multi-level approach can be effective in increasing PA in both adults [15] and children [16]. However, none of the studies included in these reviews specifically targeted families at risk for the development of T2DM [15, 16].

Therefore, the Feel4Diabetes (F4D)-intervention was developed, using a theoretical framework based on the PRECEDE-PROCEED model. More details on the development of the intervention can be found elsewhere [17]. The intervention aimed to promote a healthy lifestyle through the provision of a more supportive social and physical environment on different levels (i.e. family, school and community) to prevent T2DM in vulnerable families (i.e. living in low socioeconomic municipalities) in Europe [17]. One of the main aims of the intervention was to increase PA in families. As it is expected that changes in important correlates of PA occur before changes in PA [18], the intervention also targeted several of these correlates. More specifically, correlates targeted in adults were perception of body weight, social influence, perceived barriers, self-efficacy and knowledge, as these factors show an association with PA [19–23]. Correlates targeted in children were parental perception of body weight, parental support, attitude perceived by parents and parental knowledge, as these factors are correlated to children’s PA [24, 25].

The aim of the present study was two-fold. The first aim was to investigate the effectiveness of the F4D-intervention on the targeted correlates of PA in both adults and children of families at risk for type 2 diabetes. The second aim was to investigate the effectiveness of the F4D-intervention on PA of the study population. It was hypothesized that there would occur positive changes in the correlates in both adults and children of the intervention group between the baseline and posttest, while there would not be any changes in the correlates in the control group. In addition, due to the changes in the correlates, changes are expected in PA in the intervention study group between the baseline and the posttest and not in the control group.

## Methods

### Study design

The Feel4Diabetes study is described in detail elsewhere [17]. In short, the study implemented a school- and community-based, family-involved intervention to promote a healthy lifestyle for the prevention of T2DM among vulnerable families (i.e. families living in low socioeconomic neighborhoods). The Feel4Diabetes-intervention was tested using a cluster randomized controlled design including intervention and control families across six European countries (i.e. Bulgaria, Hungary, Belgium, Finland, Spain, Greece). To optimize cultural adaptation and increase chances for sustained implementation each country locally adapted the intervention. Furthermore, in Belgium, PA was objectively measured with Actigraph accelerometers, while in other countries other measurement tools were used (e.g. Traxmeet), which limits comparability of PA data. For the present study, only the Belgian intervention was evaluated.

**Fig. 1:**
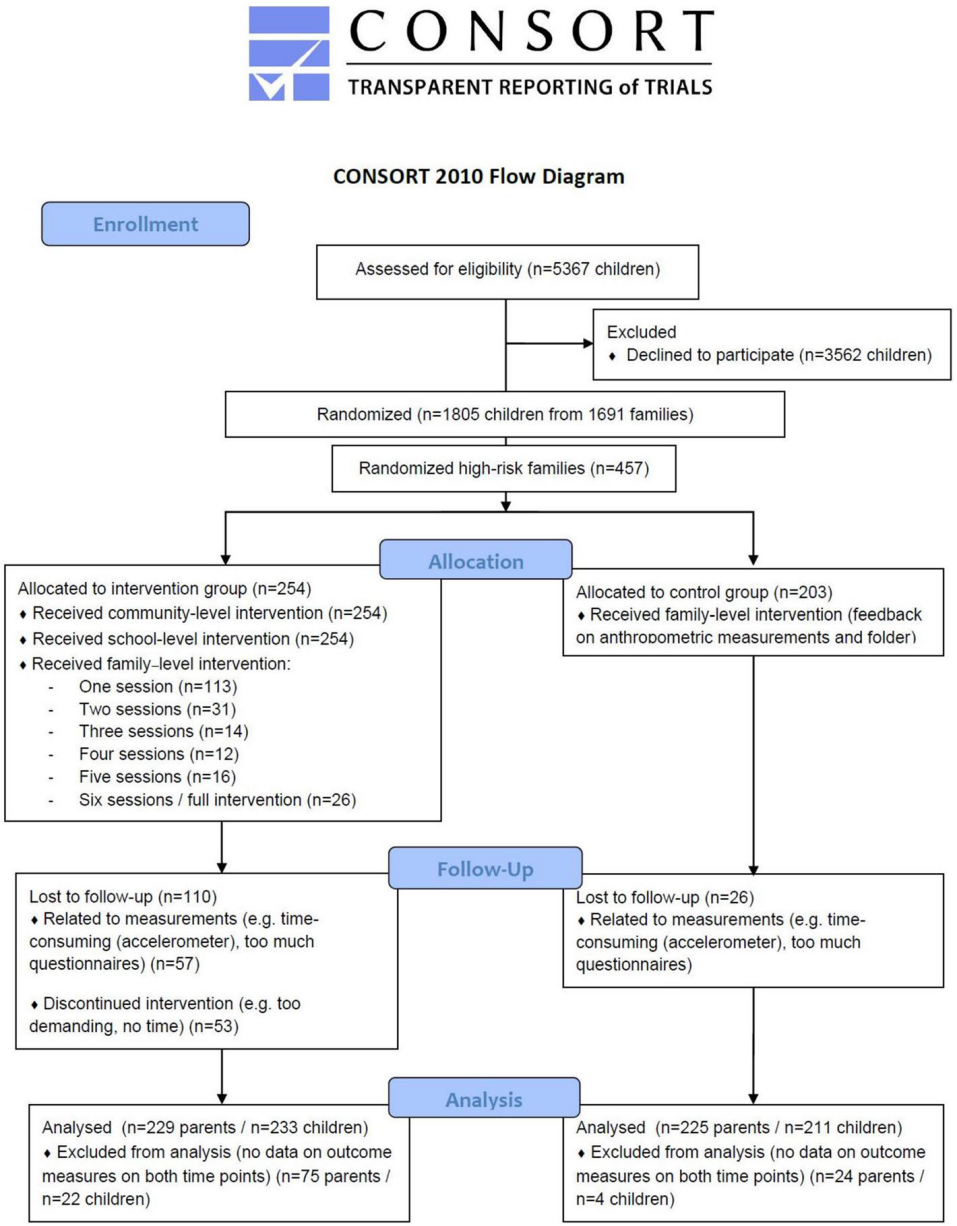
Flow chart of included parents and children with data on both baseline and follow-up test

For the recruitment of young families (children of 1st, 2nd and 3rd grade and their parent(s)), a standardized, multistage sampling approach was used. More details on the recruitment strategy can be found in the study of Manios et al. (2018) [17]. Power analyses were performed before the start of the study, and were based on the primary outcome measure in the Feel4Diabetes-study, i.e. Body Mass Index. For the high-risk families, the analyses showed that a sample size of at least 150 families per country would be sufficient to achieve sufficient statistical power (greater than 80%, at a two-sided 5% significance level) for reducing BMI by 0.7 kg/m^2^ in the high risk adults. Every country had to recruit 150 families (at least 300 adults) in the high-risk component of the intervention, and another 150 families (at least 300 adults) in the control group. To account for a possible drop-out of 20%, an additional 60 families were recruited [17]. In Flanders (Belgium), 11 municipalities from the tertile with the highest unemployment rates (5.2–12.5%) were randomly selected. Within the municipalities there was participation of 58 primary schools (response rate = 62.4%). Of all invited families (children of first to third grade (6–9 years old) and their parent(s)), 1691 families (response rate = 33.5%) confirmed their participation in the study by completing the informed consent, the Finnish Diabetes Risk Score (FINDRISC, assessing the 10-year risk of developing T2DM [26]) and the Energy Balance-Related Behavior questionnaire (EBRB-questionnaire) (see Fig. 1). Of these families, 457 families were identified as high-risk (27.0%) (i.e. at least one parent with an increased risk of developing T2DM based on the score on the FINDRISC). After the confirmation of schools and families, municipalities were assigned to the intervention (three municipalities) and the control group (eight municipalities), based on the number of inhabitants in the municipalities.

Between April and June 2016, anthropometric measurements (height, weight) were performed on all participating children by researchers during a school visit. High-risk families (both the child and one parent) were asked to wear accelerometers for five consecutive days and to complete two extra questionnaires (one on children’s health behavior and correlates and one on parents’ health behavior and correlates) (i.e., high-risk questionnaires). Between April and September 2016, high-risk parents were visited at home by a researcher for anthropometric measurements (height, weight, waist circumference, blood pressure) and blood sampling. For the present study, only high-risk families (parents and their children) were analyzed.

### Feel4Diabetes intervention

The Feel4Diabetes intervention was implemented over two school years (2016–2017 and 2017–2018) and involved three different components: (1) the family component, (2) the school component and (3) the community component. The school and community components were organized for all participating families (i.e. high-risk and non-high-risk families). The family component was organized for the high-risk families only. The first intervention year in Belgium will be described in detail below. The second intervention year focused on maintenance of the behavior change of participants, using an SMS-intervention on family level.

#### Family component, for high-risk families

High-risk families of the intervention group were invited to participate in six counseling sessions (two individual and four group sessions), that took place in their child’s school or in a school nearby. During these sessions, a trained health professional encouraged participants to adopt a healthier lifestyle (i.e. healthy eating, improving PA and limiting sedentary behavior; see Table 1) and families set SMART-goals (Specific, Measurable, Attainable, Realistic and Timely). Families of the control group only received the first individual session.

**Table 1:** Topics and content of counseling sessions

Session (period)	Topic	Content	Practical tools, activities and homework tasks
1 – individual session (September–October 2016)	Healthy lifestyle	• Feedback on anthropometric measurements and blood analysis • Folder with general information regarding healthy breakfast, healthy snacking, drinking water, proportions of food groups on a plate, PA, sedentary behavior, healthy weight	
2 – group session (November 2016)	Diabetes and risk factors	• Content and goals of the Feel4Diabetes-project • Information on T2DM and its risk factors • Quiz on healthy lifestyle	• Quiz on risk factors for T2DM • Practical assignment on sugar in beverages • Wearing pedometer for a week • Diary on PA and sedentary behavior
3 – individual session (December 2016)	SMART-goals	• Analysis of current lifestyle • Identification of goals • SMART-goal formulation • Pitfalls and solutions	
4 – group session (January 2017)	PA and sedentary behavior	• Reviewing SMART-goals • Definition and recommendations of PA and sedentary behavior • Benefits of sufficient PA and limited sedentary behavior • Parental skills regarding PA and sedentary behavior in video format	• Reviewing of diaries on PA and sedentary behavior and exchange of tips to be more physically active or be less sedentary • Perception on own PA and children’s PA with movement break based on the answer • Suggestions for apps on sedentary behavior • Diary on eating habits
5 – group session (February 2017)	Healthy eating behavior	• Healthy breakfast • Reviewing SMART-goals • Information regarding healthy breakfast, healthy snacking, meals, drinks, grocery shopping (food labeling) and mindful eating • Parental skills regarding healthy eating behavior	• Portion size task • Reward system with stickers • Task on reading food labels • Reviewing of diary on eating habits • Cookbook • Calendar on seasonal vegetables
6 – group session (March 2017)	Interactive game	• Reviewing SMART-goals • Interactive game with questions on healthy behaviors discussed in previous sessions	

#### School component

In September–October 2016, a meeting was held with the head masters and teachers from all participating intervention schools. Researchers gave suggestions and examples of activities to promote children’s PA (e.g. markings on the playground), healthy snacking (e.g. implementing a fruit day), drinking water (e.g. a water station in the classroom) and reducing sedentary behavior (e.g. movement breaks) in the school context. These activities were described in a teachers’ guide. Additionally, an overview of ongoing activities and some goals and specific plans for the upcoming academic year was created. Through the schools, all participating parents received a brochure with tips on a healthy lifestyle (i.e. sufficient PA, healthy diet, reducing sedentary behavior and water consumption). The control group did not receive an intervention on the school level.

#### Community component

Existing health-related activities in the intervention communities that proved to be suitable for the target group (e.g. inexpensive, non-competitive, for a young public (children and/or their parents), etc.) were bundled in monthly community-specific activity calendars. The calendars were sent to the schools for promotion in the classroom and were uploaded on community-specific Facebook pages. On the Facebook pages, information regarding a healthy lifestyle was also provided (e.g. newspaper articles, videos regarding the importance of reducing sitting time, etc.). The control group did not receive an intervention at the community level.

### Measurements

Measurements were performed at baseline (April–September 2016) and after 1 year (March–August 2017).

#### Demographic variables

Demographic variables of both parents and children were reported in the EBRB-questionnaire and both high-risk questionnaires. Demographic variables of parents included age, gender, ethnic background, employment status and educational level (years of education), which was used as proxy for individual socioeconomic status (SES). Demographic variables of children included age, gender and SES (based on the educational level of the mother). Educational level was dichotomized in low (having no higher education) and high SES (having higher education), ethnic background was dichotomized in Caucasian and non-Caucasian and employment status was dichotomized in employed and unemployed for analyses.

#### Attendance rates

Attendance rates of the high-risk intervention families were reported by research assistants during the counseling sessions.

#### BMI

In parents and children, body mass index (BMI) (kg/m^2^) was calculated based on objectively measured weight and height. In children, BMI z-scores were calculated based on z-score calculation of the World Health Organization (WHO; observed value of the reference population / standard deviation value of reference population) [27].

#### Diabetes risk

The FINDRISC-questionnaire, a validated tool for predicting the risk of T2DM [26], was used to assess parental diabetes risk. A score of nine or more is often used to identify parents at high-risk, as this score could identify more than 70% of incident cases of T2DM [28]. In Belgium, adults with a cut-off point of nine were included if their answers on the FINDRISC indicated an unhealthy lifestyle (e.g. did not reach 30 min of PA every day, did not eat fruit and vegetables every day or had a waist circumference that indicates a risk of metabolic complications, based on the criteria of WHO [29]).

#### Physical activity

The valid and reliable Actigraph accelerometers (GT1M, GT3X, GT3X+) were used to measure PA of parents and children [30–32]. Participants were asked to wear the accelerometers for five consecutive days, including two weekend days. Information letters with instructions on how to handle the accelerometer, were distributed to children’s parents. Accelerometers were attached at the right hip and secured by an elastic belt. Participants attached the accelerometer when they woke up in the morning and removed it when going to sleep and for water-based activities. Parents’ and children’s accelerometers were set to measure PA in epochs of respectively 1 min [33, 34] and 15 s [35]. ActiLife version 6.13.3 (Actigraph, Fort Walton Beach, FL, USA) was used to clean and score the data. Non-wear time was defined as > 60 min (parents) [36] and > 20 min (children) [37] of consecutive zero counts per minute. To score the data, accelerometer cut-points of Freedson [38] and Evenson [39] were used for parents and children respectively. For both parents and children, minutes of light PA (LIPA), minutes of moderate to vigorous PA (MVPA) and steps per day were assessed for all days and for week and weekend days separately, adjusted for non-wear time. Data from parents with at least 10 h of wearing time [36] and from children with at least 6 h of wearing time for at least 4 days (with a minimum of one weekend day) [40] were included in the analyses.

#### Correlates

For both parents and children, correlates were assessed in the high-risk questionnaires. Perception of body weight, perceived social influence, perceived barriers, self-efficacy and knowledge were assessed for parents. ***Perception of body weight*** was assessed using the question ‘What is your opinion about your current body weight?’ (five-point scale, ranging from ‘I am underweight’ to ‘I am overweight’). The question ‘How much do significant others motivate you to be physically active?’ assessed ***perceived social influence*** (five-point scale, ranging from ‘a lot’ to ‘never’; two items, Cronbach’s alpha (α) =0.90). Participants were asked ‘How likely are you to say you do not exercise because of given reasons?’ to investigate ***perceived barriers*** (four-point scale, ranging from ‘very likely’ to ‘not likely at all’). After conducting factor analyses, three scales were constructed: *environmental barriers* (four items: neighborhood lacks facilities appropriate for walking; no suitable facilities; neighborhood lacks aesthetics and pleasantness to walk or exercise; neighborhood is not safe, α = 0.79), *attitudinal barriers* (three items: too lazy/not motivated to be physically active; do not enjoy PA; never keep up a work out, α = 0.83) and *barriers concerning time constraints* (two items: no spare time; other interesting things to do, α = 0.49). The question ‘How confident are you to be physically active under the given situations?’ (e.g. during holidays, when you are anxious) (ten-point scale, ranging from ‘totally not confident’ to ‘very confident’; six items, α = 0.86) assessed ***self-efficacy***. ***Knowledge*** was assessed using the question ‘How many minutes do you think an adult should be active each day?’ (multiple-choice format: 10 min/day, 15 min/day, 20 min/day, 30 min/day, 45 min/day, 60 min/day, ‘I do not know’).

Parental perception of the weight of their child, parental support, attitude perceived by parents and parental knowledge were assessed for children. All questions were answered by one of the child’s parents. For ***perception of body weight***, parents were asked what they thought about their child’s current body weight (five-point scale, ranging from ‘he/she is underweight’ to ‘he/she is overweight’). ***Parental support*** was assessed with five items. For three items, parents were asked how often they acted in a certain way (e.g. ‘How often does at least one parent/caretaker encourage your child to participate in a movement activity or game?’; five-point scale, ranging from ‘very often’ to ‘never’) and for the two other items, parents were asked to what extent they agreed with two statements (e.g. ‘My child can skip movement activities or planned sports lessons whenever he/she wants’; five-point scale, ranging from ‘disagree’ to ‘agree’). The five items were combined in one scale (α = 0.83). To assess the ***attitude*** of the child, the question ‘To what extent do you agree with the statement ‘My child prefers watching TV or reading a book over being active’?’ was used (five-point scale, ranging from ‘disagree’ to ‘agree’). ***Knowledge*** was assessed by the question ‘How many minutes do you think children should be active each day?’ (multiple-choice format: 15 min/day, 30 min/day, 45 min/day, 60 min/day, 90 min/day, 120 min/day, ‘I do not know’).

To assess test-retest reliability of the correlates, parents who had similar demographic characteristics as the targeted population in the Feel4Diabetes-intervention completed the questionnaires twice, within a 1–2 week interval in January–March 2016. Mean intraclass correlation coefficient (ICC) for the correlates of parents was 0.80 and ranged from ICC 0.13 (‘I do not exercise because my neighborhood lacks sidewalks, bicycle lanes, parks or pavements appropriate for walking’) to ICC 0.91 (‘How confident do you feel that you can continue being physically active even when you feel depressed?’). Test-retest reliability of the correlates of children ranged from ICC 0.69 (‘How often does at least one parent/caretaker encourage your child to participate in a movement activity or game’) to ICC 0.89 (‘What is your opinion on the current body weight of your child?’), with a mean ICC of 0.82.

### Statistical analyses

Tests for normal distribution on all outcome measures revealed skewed light PA (LIPA), moderate to vigorous PA (MVPA) and daily steps for both parents and children. Therefore, logarithmic transformations were conducted for these variables. Sample characteristics and attendance rates were described and differences in characteristics between intervention and control group were investigated using respectively descriptive statistics and sample t-test and chi-square tests in SPSS 24.0 for Windows. To compare participants who had valid data (i.e. data on at least one of the outcome variables) on both time points (baseline and follow-up) and participants who did not have valid data on both time points, attrition analyses were conducted as a logistic regression with 4 levels (participant, class, school and municipality) in MLwiN 3.02 (Centre for Multilevel Modelling, University of Bristol, UK).

To assess the effectiveness of the Feel4Diabetes-intervention on parents’ and children’s PA levels and correlates and to control for clustering of participants in classes, schools and municipalities, multilevel repeated measures analyses were conducted using MLwiN 3.02. The two-way interaction effect of ‘time x group’, with five levels (time, participant, class, school and municipality) was considered for parents and for children. As ‘time’ was included as a level, all participants with valid data on at least one of both time points (454 parents and 444 children) were included in the analyses. Analyses for parents were adjusted for age, gender, SES, ethnic background, employment status, BMI and FINDRISC-score. Analyses for children were adjusted for age, gender, SES and BMI z-scores. For all analyses, statistical significance level was set at *p* <  0.05.

To calculate effect sizes of significant interaction effects, the proportion of variance explained by the interaction variable time x group in addition to other variables (η_p_^2^) was used. Explained variances of 2% or smaller, 12–25 and 26% or higher identified a small, medium or large effect size respectively. The formula used for the calculation of the effect sizes is the total variance of the model without the interaction term minus the total variance of the full model, divided by the total variance of the model without the interaction term ((Model_WithoutInteraction_ - Model_Full_) / Model_WithoutInteraction_). To obtain an effect size in percentages, the outcome was multiplied by 100. Effect sizes are only reported in the text and not in the table.

## Results

### Sample characteristics

Attrition analyses in parents showed differences in SES, ethnic background and diabetes risk score. Parents with valid data on both time points were more likely to be higher educated (OR (odds ratio)_SES_ = 1.14, 95% CI (confidence interval)_SES_ = 1.04, 1.25), Caucasian (OR_ethnicbackground_ = 1.27, 95% CI_ethnicbackground_ = 1.05, 1.50) and have a higher diabetes risk score (OR_FINDRISCscore_ = 1.14, 95% CI_FINDRISCscore_ = 1.02, 1.25). Attrition analyses in children showed differences in SES. Children with valid data on both time points were more likely to have a higher SES (OR_SES_ = 1.69, 95% CI_SES_ = 1.22, 2.16). Baseline sample characteristics of parents and children can be found in Table 2.

**Table 2:** Sample characteristics of participants in Belgium

**PARENTS**				
	**Total** **(*n*** **= 545)**	**Control group** **(*n*** **= 225)**	**Intervention group** **(*n*** **= 229)**	***p*-value**
Age (years (± SD))	39.4 (± 5.3)	39.4 (± 5.3)	39.5 (± 5.4)	0.90
Gender (% women)	72.0	72.0	72.1	0.99
SES (% high)	54.7	56.5	52.8	0.46
Ethnic background (% Caucasian)	90.7	97.9	83.6	< 0.001
Employment status (% employed)	85.0	87.1	83.0	0.25
BMI (kg/m^2^ (± SD))	27.6 (± 5.1)	27.2 (± 4.8)	27.9 (± 5.4)	0.18
FINDRISC-score (± SD)	9.6 (± 4.3)	9.5 (± 4.3)	9.6 (± 4.4)	0.95
MVPA-recommendation on all days (% achieving)	62.9	64.9	62.3	0.95
MVPA-recommendation on weekdays (% achieving)	72.8	75.9	72.9	0.86
MVPA-recommendation on weekend days (% achieving)	37.4	38.0	36.0	0.64
Step-recommendation on all days (% achieving)	13.0	13.3	12.1	0.75
Step-recommendation on weekdays (% achieving)	22.2	26.8	19.9	0.36
Step-recommendation on weekend days (% achieving)	17.6	18.9	16.0	0.23
**CHILDREN**				
	**Total** **(*n*** **= 444)**	**Control group** **(*n*** **= 211)**	**Intervention group** **(*n*** **= 233)**	**p-value**
Age (years (± SD))	8.04 (± 0.9)	8.00 (± 0.9)	8.08 (± 0.95)	0.41
Gender (% girls)	50.1	49.5	50.7	0.81
SES (% high)	58.2	59.4	57.0	0.61
BMI z-scores (± SD)	0.46 (± 1.0)	0.41 (± 0.9)	0.50 (± 1.08)	0.31
MVPA-recommendation on all days (% achieving)	70.0	72.2	67.6	0.25
MVPA-recommendation on weekdays (% achieving)	68.0	65.4	69.9	0.67
MVPA-recommendation on weekend days (% achieving)	59.7	63.3	56.5	0.32
Step-recommendation on all days (% achieving)	27.5	29.4	24.8	0.09
Step-recommendation on weekdays (% achieving)	38.2	34.7	41.4	0.67
Step-recommendation on weekend days (% achieving)	19.9	13.4	16.5	0.17

*SD* standard deviation

At baseline, the mean age of the parents was 39.4 years (Min = 27.1; Max = 68.6) and the sample consisted of 72.0% women. Of all parents, 54.7% had higher education, 90.7% identified themselves as Caucasian and 85.0% were employed. Parents’ mean BMI was 27.6 kg/m^2^ and mean diabetes risk score was 9.6. Although municipalities with the highest unemployment rates were randomly selected, the included sample had higher education and employment rates compared to Belgian census data [41]. Analyses showed a difference in ethnic background between control and intervention group with less participants identifying themselves as Caucasian in the intervention group (*p* <  0.001).

At baseline, the sample of children had a mean age of 8.0 years (Min: 6.1; Max = 12.5) and consisted of 50.1% girls. Of all children, 58.2% had a higher SES and the mean BMI z-score of the children was 0.46.

### Attendance rates

High-risk intervention families attended an average of 2.4 sessions. As the first session (i.e. feedback on the anthropometric measurements and blood analysis and the folder with general information on a healthy and active lifestyle) was a session via mail, all families received this first session. Of all intervention families, 113 families (53.3%) only received the first session, 31 families (14.6%) attended two sessions, 14 families (6.6%) attended three sessions, 12 families (5.7%) attended four sessions, 16 families (7.5%) attended five sessions and 26 families (12.3%) attended all sessions.

### Intervention effects

Results for parents are shown in Table 3. No significant intervention effects for parents were found.

**Table 3:** Intervention effects for physical activity and its correlates of parents

			Interaction effect	
	Baseline	Follow-up	Time x Group(β (SE))	95% CI
MVPA on all days (min)				
Control	42.12	43.18	0.04 (0.07)	−0.09; 0.16
Intervention	43.74	45.24		
LIPA on all days (min)				
Control	365.80	338.43	0.02 (0.02)	−0.01; 0.06
Intervention	340.20	333.24		
Steps on all days				
Control	8336.40	7748.91	0.01 (0.03)	−0.05; 0.07
Intervention	8288.19	7936.69		
MVPA on weekdays (min)				
Control	50.12	50.35	0.10 (0.07)	−0.04; 0.23
Intervention	45.49	52.62		
LIPA on weekdays (min)				
Control	360.38	332.90	0.01 (0.02)	−0.04; 0.05
Intervention	328.07	314.86		
Steps on weekdays				
Control	9564.35	8923.87	0.02 (0.03)	−0.04; 0.08
Intervention	8936.84	8865.95		
MVPA on weekend days (min)				
Control	29.18	30.03	−0.04 (0.10)	−0.23; 0.15
Intervention	39.20	32.93		
LIPA on weekend days (min)				
Control	361.97	334.53	0.03 (0.03)	−0.02; 0.08
Intervention	354.97	349.54		
Steps on weekend days				
Control	6885.49	6279.98	0.01 (0.04)	−0.07; 0.09
Intervention	7798.52	6947.57		
Perception of body weight (on 5)				
Control	3.63	3.63	0.18 (0.09)	−0.00; 0.37
Intervention	3.60	3.79		
Social influence (on 5)				
Control	3.21	3.29	−0.19 (0.17)	−0.52; 0.13
Intervention	3.11	2.99		
Environmental barriers (on 5)				
Control	3.57	3.50	−0.06 (0.07)	−0.19; 0.07
Intervention	3.56	3.43		
Attitudinal barriers (on 5)				
Control	3.23	3.26	−0.02 (0.08)	−0.18; 0.15
Intervention	3.22	3.23		
Time barriers (on 5)				
Control	2.86	2.85	−0.03 (0.10)	−0.24; 0.17
Intervention	2.81	2.77		
Self-efficacy (on 10)				
Control	6.20	5.95	0.11 (0.28)	−0.44; 0.65
Intervention	6.07	5.93		
Knowledge (on 1)				
Control	0.19	0.21	0.04 (0.08)	−0.12; 0.19
Intervention	0.23	0.28		

*p < 0.05

Results for children are shown in Table 4. There were significant negative intervention effects for MVPA on weekdays (β (SE) = − 0.06 (0.03), *p* = 0.046, η_p_^2^ = 0.80%) and steps on weekdays (β (SE) = − 0.04 (0.02), *p* = 0.029, η_p_^2^ = 0.56%). MVPA on weekdays in the intervention group decreased (− 10.87 min), while MVPA increased (+ 3.35 min) in the control group going from baseline to follow-up. Steps per day on weekdays decreased more in the intervention group (− 1385.85 steps) than in the control group (− 27.54 steps). No other significant intervention effects were found.

**Table 4:** Intervention effects for physical activity and its correlates of children

			Interaction effect	
	Baseline	Follow-up	Time x Group(β (SE))	95% CI
MVPA on all days (min)				
Control	73.30	76.93	−0.04 (0.03)	−0.10; 0.02
Intervention	73.54	66.20		
LIPA on all days (min)				
Control	285.43	271.82	0.00 (0.01)	−0.03; 0.03
Intervention	287.05	260.02		
Steps on all days				
Control	11,550.69	11,757.17	−0.03 (0.02)	−0.06; 0.01
Intervention	11,427.41	10,708.73		
MVPA on weekdays (min)				
Control	73.76	77.11	−0.06 (0.03)*	−0.12; − 0.00
Intervention	77.21	66.34		
LIPA on weekdays (min)				
Control	291.19	274.11	−0.00 (0.01)	−0.03; 0.02
Intervention	302.26	271.57		
Steps on weekdays				
Control	12,089.45	12,061.91	−0.04 (0.02)*	−0.08; − 0.00
Intervention	12,384.86	10,999.01		
MVPA on weekend days (min)				
Control	72.78	76.20	−0.02 (0.05)	−0.12; 0.08
Intervention	67.79	66.18		
LIPA on weekend days (min)				
Control	275.88	261.20	0.01 (0.02)	−0.03; 0.05
Intervention	258.68	237.79		
Steps on weekend days				
Control	10,738.78	11,030.24	0.01 (0.03)	−0.06; 0.08
Intervention	9915.47	10,155.25		
Perception of body weight (on 5)				
Control	2.98	2.95	0.08 (0.08)	−0.07; 0.23
Intervention	2.84	3.10		
Parental support (on 5)				
Control	3.71	3.63	0.09 (0.09)	−0.08; 0.26
Intervention	3.60	3.71		
Attitude (on 5)				
Control	3.63	3.46	0.20 (0.20)	−0.18; 0.59
Intervention	3.66	3.31		
Knowledge (on 1)				
Control	0.42	0.51	0.02 (0.08)	−0.14; 0.19
Intervention	0.48	0.62		

*p < 0.05

## Discussion

This study aimed to investigate the effect of the first year of the Feel4Diabetes-intervention on PA and correlates in vulnerable families (i.e. families at risk for T2DM and living in low socio-economic municipalities) in Belgium. Although the intervention used a multi-level approach, which has proven effective in increasing PA previously [15, 16, 42], we found few significant intervention effects. The intervention expected to see changes of children’s PA or correlates through changes in parental PA or correlates, as it has been proven that parents’ role modeling and parental support play an important role in PA behavior in children [43, 44], but results did not show these expected changes. Children’s MVPA and steps per day on weekdays changed significantly, but interestingly decreased (more) in the intervention group. However, effect sizes showed no biologic relevance of these results.

First, the lack of intervention effects in parents could explain the lack of positive intervention effects in children. The lack of effects in parents could be due to the fact that, on average, parents of both intervention and control group already achieved the recommended PA levels [45] at baseline (i.e. 43.74 min and 42.12 min of MVPA/day, respectively). Results of the study also show that 62.3 and 64.9% of parents in the intervention and control group, respectively, achieved the recommended PA levels. These are unexpected results as evidence states that insufficient PA in adults is more prevalent in low socio-economic areas [46], but could be explained by the higher individual SES of participating parents. Second, on average, also PA-levels in children on weekdays were already high at baseline (i.e. 68% of children already achieved MVPA-recommendations [47]). Third, the negative intervention effect in children could partially be explained by the fact that only parents and teachers were actively involved in the components and children were only involved in one of the counseling sessions.

The overall lack of positive effects of the Feel4Diabetes-intervention on PA and its correlates in vulnerable families in Belgium could be due to low attendance rates of families at the counseling sessions and the large drop-out at follow-up. The reports showed that only 26 families attended all session. Thorough drop-out tracking showed that parents and children who dropped out of the study were more likely to have a lower SES, which reflects evidence stating that it is more difficult to reach vulnerable populations [48]. Therefore, future interventions targeting vulnerable populations should look for more effective strategies to address and retain these groups. First of all, it seems important to use face-to-face recruitment within the community [48]. Second, it will be important to target the individual together with its social network, as one of the key influences on behavior change within low SES groups is social support [49]. Lastly, development of interventions focusing on improving physical activity in vulnerable populations should be more bottom-up and interventions should target activity-related attitudes and skills more [50].

The first important strength of this study is that it targeted vulnerable families, who remain an important target group in T2DM-prevention as they have a higher risk of developing the disease [8, 9]. Targeting these groups could help reduce health inequalities [51, 52]. A second strength of this study is the objectively measured PA levels of parents and children by using accelerometers. The current study also has some limitations. First, a lot of participants had incomplete data, which could limit conclusions on causality and results should be interpreted with caution. In addition, attrition analyses showed that parents with valid data were more likely to be higher educated, Caucasian and have a higher risk score and children with valid data were more likely to have a higher SES. Consequently, results should even be more interpreted with caution. Second, the second intervention year, which focused on maintaining the behavior change, was not taken into account in the analyses, while perhaps more time is needed to really change lifestyle behavior. Third, attendance at the counseling sessions was very low, which could have limited the impact of the intervention on participants that did not attend the sessions. Fourth, children were not actively involved in the intervention, which could have caused the limited effect of the intervention on children’s PA. Fifth, there is a lack of communication on more in-depth process evaluation data, such as intervention reach, dose and fidelity. Consequently, it is unknown if the intervention was implemented correctly and consistently. If not, this could help in explaining the null or even negative intervention effects. And last, generalizability of the results is limited to a very specific group of the population, namely members of families with an increased risk on type 2 diabetes from low socio-economic regions in Flanders. Furthermore, the intervention was mainly performed in urban municipalities and as a result, the generalizability to more rural areas is unknown.

## Conclusions

The multilevel Feel4Diabetes-intervention lacks effectiveness on objectively measured physical activity and its correlates of Belgian families (i.e. children and their parent(s)) at risk for the development of type 2 diabetes. These results are mainly due to a high drop-out and low attendance rates of the participants. Therefore, future interventions should seek to integrate more effective strategies to address and retain this target group.

## Data Availability

The datasets used and/or analysed during the current study are available from the corresponding author on reasonable request.

## Abbreviations

T2DM: type 2 diabetes mellitus
PA: physical activity
F4D: Feel4Diabetes
FINDRISC: Finnish Diabetes Risk Score
EBRB: Energy Balance-Related Behavior
SMART: Specific, Measurable, Attainable, Realistic and Timely
SES: socioeconomic status
BMI: body mass index
WHO: World Health Organization
ICC: intraclass correlation coefficient
LIPA: light physical activity
MVPA: moderate to vigorous physical activity
OR: odds ratio
CI: confidence interval
SD: standard deviation
